# DECISION-AID INTERVENTIONS TO SUPPORT DEMENTIA CAREGIVERS' DECISION-MAKING ABOUT FEEDING OPTIONS

**DOI:** 10.1093/geroni/igac059.265

**Published:** 2022-12-20

**Authors:** Xiang Qi, Yaolin Pei, Dena Schulman-Green, Mengyao Hu, Kaipeng Wang, Bei Wu

**Affiliations:** New York University, New York City, New York, United States; New York University, New York City, New York, United States; Rory Meyers College of Nursing, New York University, New York City, New York, United States; New York University, New York, New York, United States; University of Denver, Denver, Colorado, United States; New York University, New York, New York, United States

## Abstract

We sought to provide an overview of the literature on decision aid interventions for family caregivers of older adults with advanced dementia regarding decision-making around tube feeding. The process was guided by Arksey and O’Malley’s methodological framework. Six publications reporting on four unique decision aid interventions were included. All the interventions targeted caregivers of older adults with advanced dementia. Three of decision aids were culturally adapted from existing ones. The Ottawa Decision Support Framework and the International Patient Decision Aid Standards Framework were used in these studies. Interventions aimed to improve decision-making regarding tube feeding for caregivers through static delivery methods. Caregivers rated these decisions as helpful and acceptable. Reduction in decisional conflict and increase in knowledge were consistently found among dementia caregivers, but no intervention effects were found on preferences for use of tube feeding. Culturally adapted decision aids effectively improve decision-making regarding tube feeding among the target population.

